# Nectin-4 expression in clear cell renal cell carcinoma

**DOI:** 10.1038/s41598-026-58406-0

**Published:** 2026-06-26

**Authors:** Kyra Klein, Steffen Rausch, Veronika Bahlinger, Moritz Maas, Simon Walz, Igor Tsaur, Viktoria Stühler

**Affiliations:** 1 Department of Urology, University Hospital Tuebingen, Eberhard-Karls-University, Tuebingen, Tuebingen, Germany; 2 Department of Pathology, University Hospital Tuebingen, Eberhard-Karls- University Tuebingen, Tuebingen, Germany

**Keywords:** Antibody-drug conjugates (ADC), Enfortumab Vedotin, Nectin-4, renal cell carcinoma, Biomarkers, Cancer, Oncology

## Introduction

Nectins mediate cell–cell adhesion via extracellular immunoglobulin-like domains. Among them, Nectin-4 is an established therapeutic target in several cancers, most notably urothelial carcinoma, where antibody–drug conjugates such as Enfortumab Vedotin (EV) bind to Nectin-4 and induce selective tumor cell death through intracellular release of a cytotoxic payload^1^. Beyond Antibody-drug conjugates (ADCs), Nectin-4 is being investigated as a target in additional therapeutic strategies, including oncolytic measles viruses that selectively infect and lyse Nectin-4–positive tumor cells^2^, as well as immunotherapeutic approaches using cytotoxic T lymphocytes directed against Nectin-4^3^. Nectin-4 also serves as a ligand for the immune checkpoint TIGIT, and its blockade may modulate T-cell and NK-cell activity^4^. Furthermore, Nectin-4–targeting CAR-T cells have demonstrated antitumor activity in preclinical models^5^. Despite these advances, Nectin-4 has received little attention in renal cell carcinoma (RCC). This study aims to characterize Nectin-4 expression in normal renal tissue, primary tumors, and metastases of clear cell RCC (ccRCC), and to evaluate its association with clinicopathological features and overall survival (OS). Ultimately, we address whether Nectin-4 expression in ccRCC supports its potential as a therapeutic target, including for ADC-based approaches.

## Materials and methods

### Patient cohort

The cohort comprised 87 ccRCC patients treated at the Department of Urology, University Hospital Tuebingen, Tuebingen, Germany, between 1992 and 2019, with follow-up until February 2023. Clinicopathological and treatment data, including OS, were recorded. Tissue from primary tumors was available for 70 patients (including 7 bilateral cases), benign renal tissue for 44, and metastatic tissue for 22; an additional 17 patients had only metastatic samples (total *n* = 53). Both written informed consent from the patients allowing the use of tissue for research purposes as well as a positive ethics vote (application number 677/2024BO2) from the Ethics Committee of the University of Tuebingen were available. A detailed overview of the clinical-pathological characteristics of the patients is shown in Table 1.

**Table 1:** Patient cohort with detailed clinicopathologic characteristics.

Characteristics of patients	*N* (%)
Histology	
Clear cell	84 (96.5%)
Clear cell and papillary	3 (3.4%)
**Available tissue**	
Primary tumor	70 (80.4%)
Primary tumor and benign tissue	44 (50.6%)
Primary tumor and metastatic tissue	22 (25.3%)
Metastatic tissue	17 (19.5%)
**Total number of metastatic tissues**	53
**Male gender**	65 (74.7%)
**Age – median (IQR)**	
at primary RCC diagnosis	62.7 (39-79)
**pT stage**	
pT1	30 (34.5%)
pT2	9 (10.3%)
pT3	44 (50.6%)
pT4	3 (3.4%)
**Grading**	
G1	7 (8%)
G2	48 (55.2%)
G3	27 (31%)
G4	1 (1.1%)
NE	4 (4.6%)
**Nodal stage at first diagnosis**	
N0	54 (62%)
N1	10 (11.4%)
N2	1 (1.1%)
N3	1 (1.1%)
NE	19 (21.8%)
**Metastasis at first diagnosis**	
M0	46 (52.9%)
M1	34 (39.1%)
NE	7 (8%)
**Metastasis**	
Synchronous	42 (48.3%)
Metachronous	45 (51.7%)
**UICC stage first diagnosis**	
I	14 (16.1%)
II	4 (4.6%)
III	22 (25.3%)
IV	32 (36.8%)
unknown	15 (17.2%)
**Resection status primary tumor**	
R0	68 (78.2%)
R1	10 (11.5%)
R2	3 (3.4%)
NE	6 (6.9%)
**Vascular invasion primary tumor**	
V0	40 (46%)
V1	29 (33.3%)
V2	7 (8%)
NE	11 (12.6%)
**Lymphativ invasion primary tumor**	
L0	71 (81.6%)
L1	4 (4.6%)
NE	12 (13.8%)
**Tumor necrosis primary tumor**	
Yes	38 (43.7%)
No	33 (37.9%)
NE	16 (18.4%)
**Sarcomatoid differentiation primary tumor**	
Yes	6 (6.9%)
No	66 (75.9%)
NE	15 (17.2%)
**Primary tumor size in cm**	
Median (range)	6.5 cm (1-18)
Mean (±SD)	7.6 (0.46)
**Fat tissue infiltration**	
No	51 (58.6%)
Perirenal	18 (20.7%)
Pelvic	5 (5.7%)
Perirenal and pelvic	3 (3.4%)
NE	10 (11.5%)
**Renal pelvis infiltration**	
Yes	9 (10.3%)
No	60 (69%)
NE	18 (20.7%)
**Capsule infiltration**	
Yes	26 (29.9%)
No	50 (57.5%)
NE	11 (12.6%)
**Adrenal gland infiltration**	
Yes	6 (6.9%)
No	71 (81.6%)
NE	10 (11.5%)
**Hilus vein infiltration**	
Yes	29 (33.3%)
No	45 (51.7%)
NE	13 (14.9%)
**Infiltration of surrounding structures**	
Yes	44 (50.1%)
No	25 (28.7%)
NE	18 (20.7%)
**BMI median (range)**	26.9 (20.3- 37.9)
**Diabetes type 2**	
Yes	22 (25.3%)
No	65 (74.7%)
**Smoking**	
Yes	20 (23%)
No	64 (73.6%)
NE	3 (3.4%)
**Hyperlipidemia**	
Yes	15 (17.2%)
No	71 (81.6%)
NE	1 (1.1%)
**Median time from primary tumor to last follow up or death**	66 (0-341)
(range, in months)	
**Median time form primary tumor to metastasis** (range, in months)	1 (0-248)
**Median follow up from primary tumor to last follow up or death (range**,** in months)**	61,5 (0-341)
**Overall survival**	
Alive	21 (24.1%)
deceased	66 (75.9%)
**Cancer-specific survival**	
Alive/ non-cancer-related death	25 (28.7%)
Cancer-related death	50 (57.5%)
unknown cause of death	12 (13.8%)
**Metastatic site distribution**	
abdominal wall	1
bronchus	1
small intestine	1
brain	3
skin	1
bone	8
liver	1
lymph node	5
local recurrence	7
lung	6
stomach	3
spleen	1
adrenal gland	4
omentum	1
pancreas	2
paratracheal	1
parotid gland	1
peritumoral fat	1
pleura	1
thoracic wall	1
soft tissue	2
diaphragm	1

Abbreviations: G grading, M distant metastasis, N regional lymph nodes, NE not evaluable, T primary tumor.

### Immunohistochemistry

Tissue microarrays (TMAs) were constructed from primary tumor tissue, benign renal tissue, and metastatic tissue. For each patient, two representative areas per tissue type were selected on eosin-stained sections, preferably central tumor regions without necrosis, larger vessels, or hemorrhage. The marked regions were identified in the paraffin block and sampled using a manual tissue microarray system (MTA-1, Beecher Instruments). Tissue cores measuring 0.6 mm in diameter and 3–4 mm in depth were extracted and transferred into a recipient paraffin block according to a predefined layout map. Two cores each from the primary tumor, benign tissue, and metastasis were included.

To date, no standardized protocol exists for Nectin-4 staining in RCC. Initial tests determined an optimal antibody dilution of 1:4000. Immunohistochemical staining was performed using an antibody against Nectin-4 (1:4000, Thermo Fisher, Waltham, MA, USA). Samples with extensive necrosis, insufficient tumor cells, or tissue loss were excluded. For quantitative evaluation of protein expression, the staining intensity of protein staining was indicated using the H-score^6^.

The H-score is calculated using the following formula:

H-Score = ((%0)x0)+ ((%1+) x1)+((%2+)x2)+((%3+)x3) with a maximum value of 300.

The H-score was recorded for tumor, metastasis, and normal tissue, then correlated with clinicopathological data and overall survival. Membranous staining was evaluated manually in a blinded manner, with only complete membranous staining being classified as positive, whereas cytoplasmic staining was analyzed using QuPath software^7^.

### Statistical analyses

OS was analyzed using descriptive statistics and Kaplan–Meier curves, with follow-up from primary tumor surgery to cancer-related death or last follow-up. Variables significant in univariate Cox regression were included in a multivariate model to assess independent prognostic value. As most variables were not normally distributed, non-parametric statistical methods were applied. Associations between variables were assessed using Spearman’s rank correlation, while paired data were analyzed with the Wilcoxon signed-rank test and differences between two independent groups with the Mann–Whitney U test. Cutoff values for Nectin-4 expression were determined based on the distribution of H-scores within the study cohort. The median H-score was used for initial dichotomization analyses. For membranous Nectin-4 expression in primary tumors, additional cutoff values were explored, and a threshold of 90 was selected for outcome analyses due to its superior discriminatory performance. Analyses were performed in SPSS, version 29, with *p* < 0.05 considered significant.

## Results

### Patient characteristics

The study included 87 ccRCC patients, three with additional papillary components, encompassing tumors from T1 (*n* = 30) and T2 (*n* = 9) to advanced pT3/4 (*n* = 47). Median follow-up was 66 months (0–341). Overall, 66 patients (75.9%) died, including 50 tumor-specific deaths, 21 (24.1%) were alive. At diagnosis, 42 patients (48.3%) had metastases, and by last follow-up, all had experienced recurrence or metastasis.

### Protein expression

**Fig. 1:**
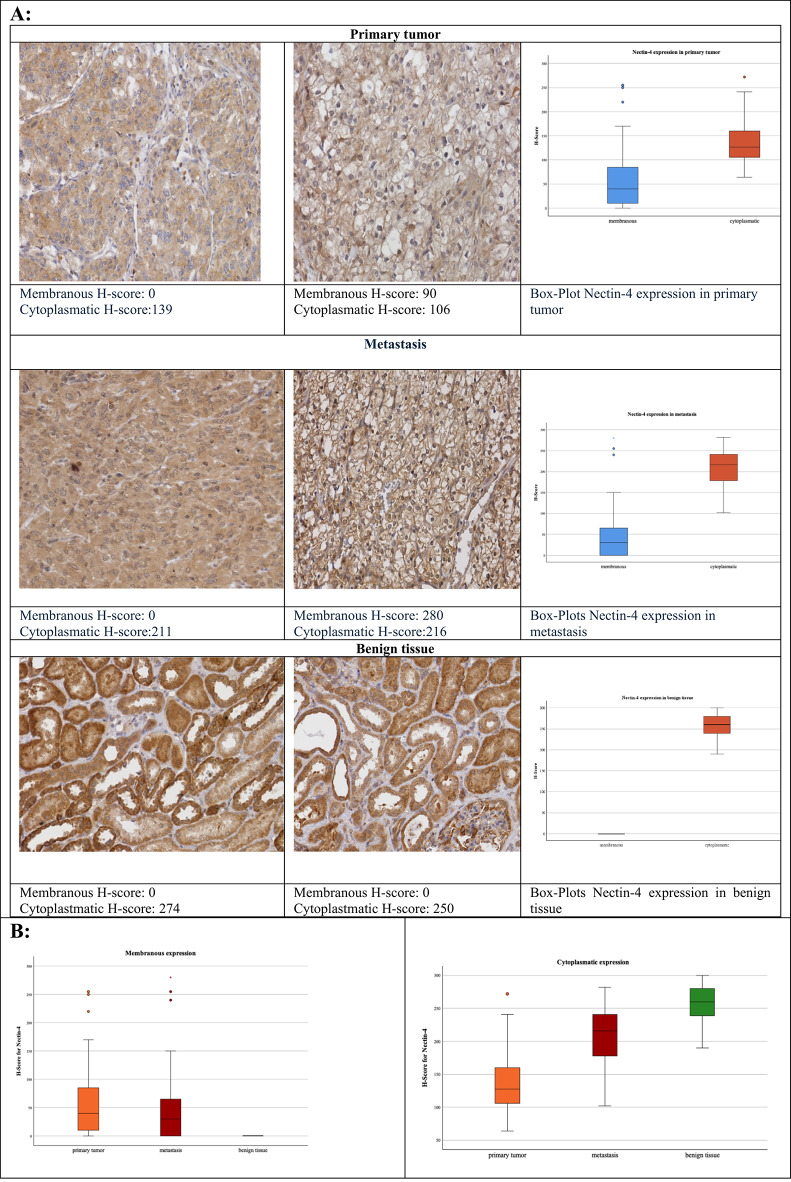

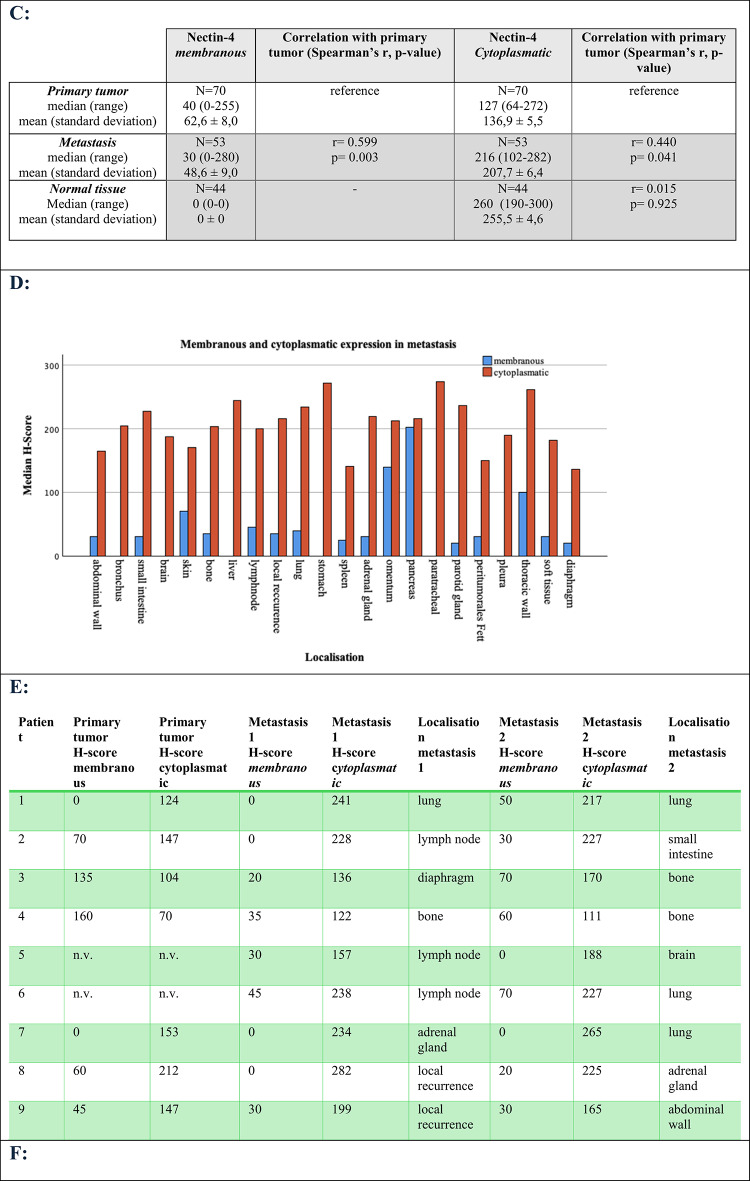

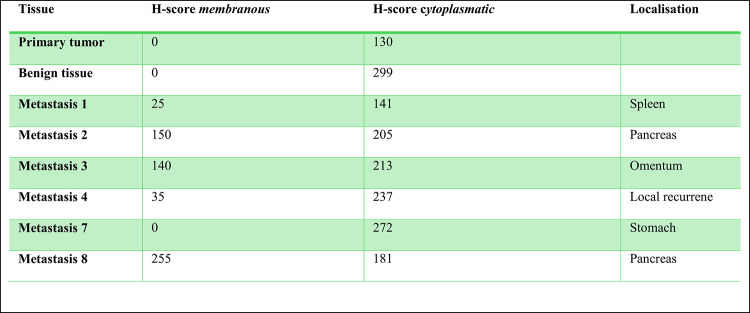
**A**: Representative immunohistochemical staining of primary tumor, metastasis and, normal tissue areas.**B**: Evaluation of Nectin-4 protein expression levels derived from the semi-quantitative scoring system and comparison between primary tumor, metastasis, and non-tumor tissue samples. **C**: Data of different protein expression levels of Nectin-4 in the primary tumor compared with metastases and with normal tissue. **D**: Nectin-4 expression by metastatic site. Expression shown as median H-score for membranous and cytoplasmic staining. **E**: Nectin-4 expression in the primary tumor and two metastases in 9 patients. **F**: H-score of Nectin-4 expression in the primary tumor, benign renal tissue and six metastases of 1 patient who was the only patient in the collective with more than two evaluable metastases. Abbreviations: m/z = membranous/cytoplasmic expression, Pt = primary tumor, Met1/2 = first/second evaluable metastasis, Lok1/2 = localization of metastases, n.a. = not available.

In benign kidney tissue, Nectin-4 was exclusively cytoplasmic, whereas primary tumors showed membranous staining in 79% and cytoplasmic staining in 100% of samples. Representative examples of the different IHC staining patterns in primary tumor, metastasis, and normal tissue are shown in Fig. 1A.

Cytoplasmic expression was generally stronger than membranous across all tissue types, being highest in normal tissue, followed by metastases, and lowest in primary tumors (mean ± SD: 255.5 ± 4.6 vs. 207.7 ± 6.4 vs. 136.9 ± 5.5; *p* < 0.001). Membranous expression was highest in primary tumors (mean ± SD: 62.6 ± 8.0), lower in metastases (48.6 ± 9.0), and absent in normal tissue. Protein expression across the different tissue types is presented in Fig. 1B. In 22 matched primary tumor–metastasis pairs, Spearman correlation analysis demonstrated that higher Nectin-4 expression in primary tumors was associated with higher expression in corresponding metastases for both membranous (*r* = 0.634, *p* = 0.003) and cytoplasmic localization (*r* = 0.440, *p* = 0.041). However, the Wilcoxon signed-rank test revealed no significant difference in membranous Nectin-4 expression between primary and metastatic lesions (Z=–1.765, *p* = 0.078).

Expression patterns across sites and over time were variable, see Fig. 1D and E, and Fig. 1F.

**Fig. 2:**
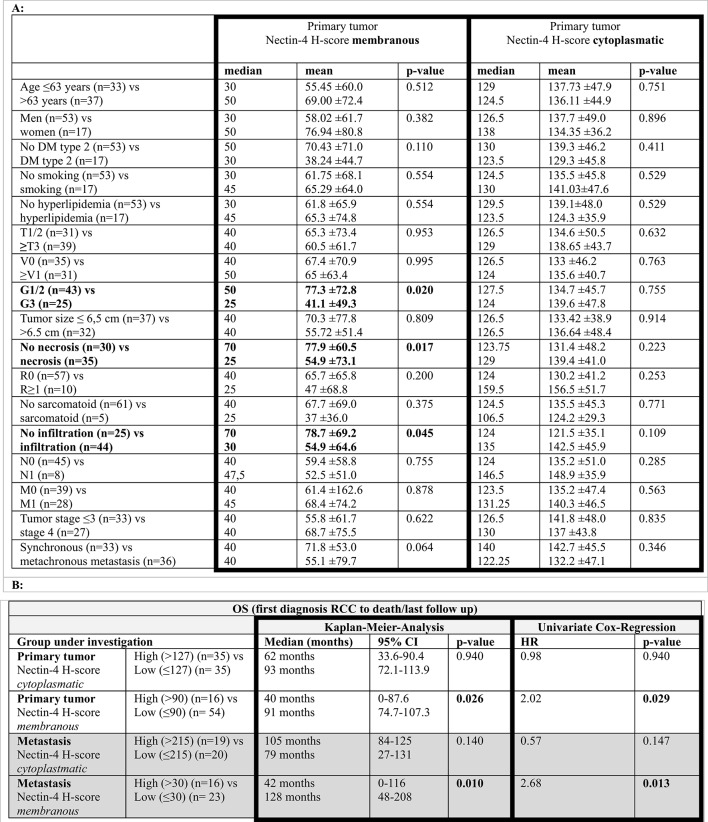

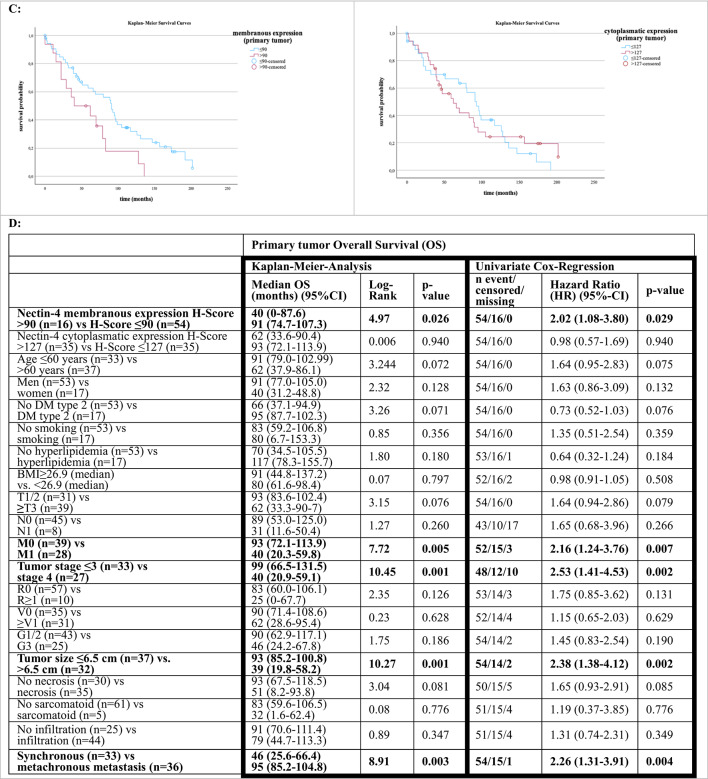

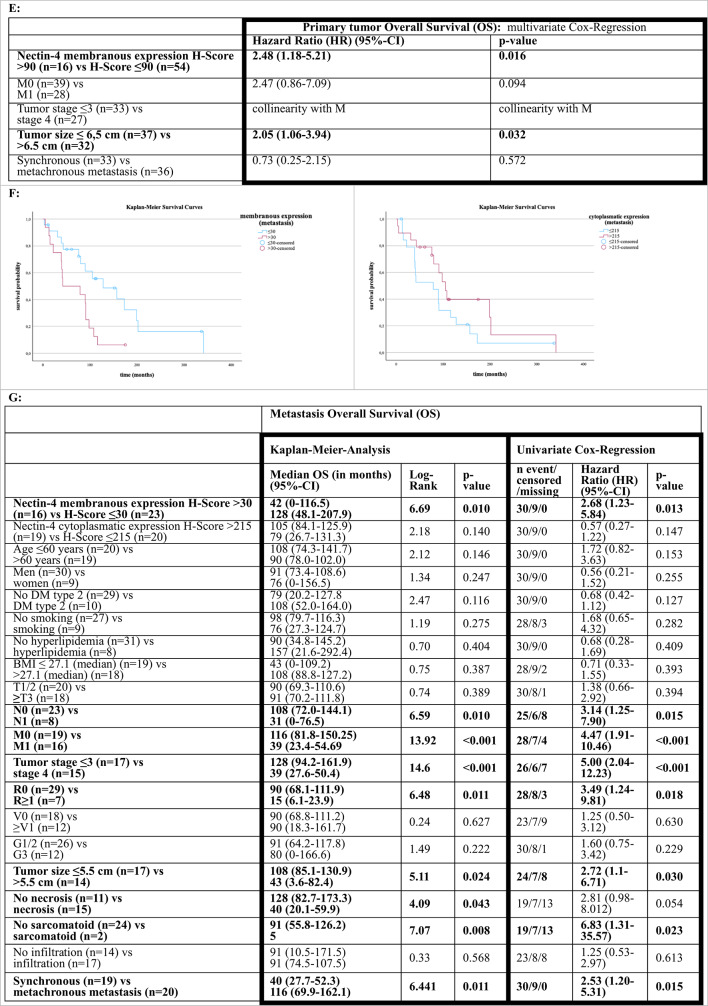

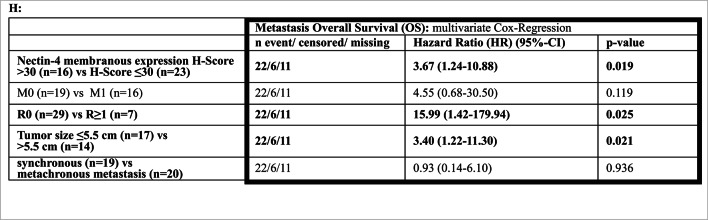
**A** Univariate analysis of protein expression for Nectin-4 **in primary tumor** depending on clinical parameters. **B** Overall Survival, defined as time from initial diagnosis to death/last follow-up in months, as a function of protein expression of Nectin-4 in the primary tumor and in metastasis. **C** Kaplan–Meier analyses for OS depending on Nectin-4 expression in primary tumors. **D** Overall survival (OS) related to clinicopathological parameters, including primary tumor Nectin-4 expression, based on Kaplan–Meier and univariate Cox regression analyses. **E** Multivariate analysis for OS as time from initial diagnosis to death/last follow-up, as a function of protein expression of Nectin-4 in primary tumor. **F** Kaplan–Meier analyses for OS depending on Nectin-4 expression in metastasis. **G** Overall survival (OS) related to clinicopathological parameters, including metastasis Nectin-4 expression, based on Kaplan–Meier and univariate Cox regression analyses. **H** Multivariate analysis for OS as time from initial diagnosis to death/last follow-up, as a function of protein expression of Nectin-4 in metastasis.

In primary tumors, membranous Nectin-4 expression was significantly higher in low-grade (G1/2) tumors (*p* = 0.020), and in tumors without necrosis (*p* = 0.017) or infiltration (*p* = 0.045, Fig. 2A). Cytoplasmic Nectin-4 showed no correlation with clinicopathological parameters. No significant associations were identified between Nectin-4 protein expression in metastatic tissue and clinicopathological parameters. The analysis included 39 patients, of whom 10 presented with multiple metastases; to ensure comparability, only the earliest evaluable metastatic lesion was considered. An overview of the correlations between Nectin-4 expression in primary tumors and clinicopathological parameters is shown in Fig. 2A.

Median OS for primary tumors was 80 months (95% CI: 55–105 months) with median OS based on Nectin-4 expression levels summarized in Fig. 2B. In univariate Cox regression, membranous Nectin-4–positive tumors (H-score > 90) had significantly worse OS (median 40 vs. 91 months, HR 2.02, *p* = 0.029), while cytoplasmic expression (H-score > 127) had no impact on OS (median 62 vs. 93 months, HR 0.98, *p* = 0.940), see Fig. 2C.

Also distant metastases at diagnosis (HR 2.16, *p* = 0.007), advanced tumor stage (HR 2.53, *p* = 0.002), tumor size > 6.5 cm (HR 2.38, *p* = 0.002), and synchronous metastases (HR 2.26, *p* = 0.004) were all significantly associated with worse OS in univariate Cox regression,. In multivariate analysis, however, only high membranous Nectin-4 expression (HR 2.48, *p* = 0.016) and tumor size > 6.5 cm (HR 2.05, *p* = 0.032) remained independent prognostic factors for OS (Fig. 2D and E).

In patients with mRCC (*n* = 39), median OS was 91 months (95% CI: 71–110 months). In univariate Cox regression high membranous Nectin-4 expression in metastases (H-score > 30) was associated with significantly shorter OS in univariate analysis (42 vs. 128 months; HR 2.68, *p* = 0.013), whereas cytoplasmic expression had no impact on survival (H-score > 215, HR 0.57, *p* = 0.147). Kaplan-Meier survival curves illustrate the survival difference between the two groups (Fig. 2F). Univariate analysis also identified lymphogenic (HR 3.14, *p* = 0.015) and distant metastases (HR 4.47, *p* < 0.001), advanced tumor stage (HR 5.00, *p* < 0.001), R1 resection (HR 3.49, *p* = 0.018), sarcomatoid differentiation (HR 6.8, *p* = 0.023), larger tumor size (> 5.5 cm; HR 2.7, *p* = 0.030), and synchronous metastases (HR 2.53, *p* = 0.015) as adverse prognostic factors for OS. In multivariate analysis, high membranous Nectin-4 expression in metastases (H-score > 30; HR 3.67, *p* = 0.019), R1 status (HR 15.99, *p* = 0.025), and tumor size > 5.5 cm (HR 3.40, *p* = 0.021) remained independent predictors of poor survival (Fig. 2F–H).

## Discussion

Unlike Nectins 1–3, which are physiologically expressed, Nectin-4 is largely restricted to malignant cells after embryogenesis and promotes tumor proliferation, lymphangiogenesis, and angiogenesis in vitro and in vivo^8–11^. Its expression serves as a prognostic biomarker in several cancers, including breast, esophageal, lung, colorectal, and pancreatic cancer^9,10,12–14^. The efficacy of EV in Nectin-4–positive non-urothelial tumors remains unknown. While VEGF-targeted tyrosine kinase inhibitors and immune checkpoint inhibitors are standard therapies in RCC, this study aimed to investigate Nectin-4 expression and its potential diagnostic and therapeutic relevance in ccRCC. To our knowledge, this is the first study addressing this question in ccRCC.

Here, Nectin-4 showed variable expression in ccRCC, with high membranous staining in 47% of primary tumors and 45% of metastases, and high cytoplasmic expression in 43% and 49%, respectively. Staining was confined to the cytoplasm and cell membrane of tumor cells, while benign renal tissue showed only cytoplasmic tubular staining at varying intensities and no nuclear expression. Some samples displayed highly heterogeneous staining patterns both cytoplasmically and membranously, whereas others showed more uniform distribution. Cytoplasmic staining was generally stronger than membranous staining across all tissue types. Cytoplasmic Nectin-4 expression was highest in benign tissue, followed by metastases and primary tumors (*p* < 0.001), whereas membranous Nectin-4 expression was highest in primary tumors, lower in metastases, and absent in benign tissue. These findings align with data from healthy breast tissue demonstrating exclusively cytoplasmic expression^15^, and from metastatic urothelial carcinoma showing reduced membranous Nectin-4 expression in metastases compared with primary tumors^16^.

Nectin-4 expression across different sites and over time was variable, showing the highest membranous levels in pancreatic and omentum metastases, though conclusions are limited by the small sample size.

In primary tumors, higher membranous Nectin-4 was significantly associated with lower grade (G ≤ 2), absence of necrosis, and no infiltration, while cytoplasmic expression showed no correlations.

In contrast, previous studies in urothelial carcinoma reported an association between high Nectin-4 expression and unfavorable prognosis, lymphatic invasion, and higher tumor grade^17^. Similarly, in breast cancer, elevated Nectin-4 expression has been linked to larger tumor size, higher grade, lymph node involvement, metastatic spread, and reduced survival^10,11,18–20^. However, a more recent study from 2025 on urothelial carcinoma of the bladder and upper urinary tract demonstrated that high predominantly membranous Nectin-4 expression was associated with more favorable clinicopathological features and improved survival, whereas predominantly cytoplasmic expression was more frequently observed in advanced disease stages^21^.

In metastases, Nectin-4 expression did not correlate with clinicopathological parameters. These findings contrast with urothelial and breast cancer, where high Nectin-4 expression is linked to poor prognosis, higher grading, lymphatic invasion, and metastasis^1,10,11,17–20,22^.

Despite the correlation of high Nectin-4 expression with favorable clinicopathological parameters, high membranous Nectin-4 expression was identified as an independent predictor of poor OS in both primary and metastatic ccRCC (multivariate analysis *p* = 0.016 and *p* = 0.019). In primary tumors, univariate analysis linked high membranous Nectin-4 (H-score > 90), distant metastases at diagnosis, advanced tumor stage, larger tumor size, and synchronous metastases to worse survival, but multivariate analysis retained only high membranous Nectin-4 (HR 2.48, *p* = 0.016) and tumor size (HR 2.05, *p* = 0.032) as independent prognostic factors. Similar results were seen in metastases, where high membranous Nectin-4 (H-score > 30), R1 status, and larger tumor size remained significant in multivariate analysis for OS.

The apparent biological discrepancy between the association of high membranous Nectin-4 expression with favorable clinicopathological features such as lower grade, absence of necrosis, and lack of infiltration and its association with poorer OS cannot be conclusively explained based on the current data. Potential explanations may include the composition of the study cohort and the relatively limited sample size. Furthermore, Nectin-4 expression may reflect additional biological mechanisms that are not fully represented by conventional pathological parameters alone.

In contrast, cytoplasmic Nectin-4 showed no impact on survival, neither in primary tumors nor in metastatic samples, suggesting that the biological and prognostic relevance of Nectin-4 may depend not only on its presenve but also on its subcellular localization.

While membranous expression may reflect the functionally active fraction involved in tumor progression and cell signaling, cytoplasmic staining may represent intracellular storage or protein turnover. Similar observations have previously been reported in breast cancer.There, membranous Nectin-4 has been linked to aggressive disease in breast cancer, while cytoplasmic expression was associated with better outcomes^15^. Supporting our findings, *M. Rabet et al.* reported that high Nectin-4 mRNA was a negative prognostic marker for metastasis-free survival and correlated positively with protein expression in 5.673 invasive breast cancer samples^10^. Similarly, high Nectin-4 expression was associated with reduced OS in esophageal cancer (*n* = 94, HR 1.75, *p* < 0.05)^12^. In pancreatic cancer, Nectin-4 correlated with Ki67 and VEGF, and its siRNA knockdown inhibited tumor cell proliferation in vitro, low Nectin-4 levels were linked to longer survival (682 vs. 426 days, *p* = 0.013)^9^. In hepatocellular carcinoma, high Nectin-4 expression also corresponded to shorter recurrence-free survival and OS^23^.

To date, no studies have examined Nectin-4 expression in ccRCC, the present cohort is therefore of particular interest as it includes primary tumors and matched metastases. In papillary RCC, Nectin-4 expression was observed in about 44.1% of cases with 48.4% of type 1 and 36.4% of type 2 pRCC, predominantly membranous, with no significant association with OS except for improved 5-year survival in type 1 tumors with high Nectin-4 expression^24,25^. Similarly, higher Nectin-4 expression was associated with better survival, lower T stage and lower pN stage in triple-negative breast cancer^13^. In chromophobe RCC, Nectin-4 positivity was rare (18.5%) and not associated with clinical parameters or survival^24^. Preliminary data from the CICERONE study (NCT05372302) in collecting duct carcinoma showed Nectin-4 expression in 41% of cases, and TROP-2 positivity in 98%, suggesting potential eligibility for Nectin-4–targeted ADC therapies, which are currently being evaluated in clinical trials (phase II RePRINT trial: NCT06302569)^26^.

Data on the efficacy and safety of EV beyond urothelial carcinoma remain limited. Several phase I trials such as EV-101 (part A) in non-urothelial carcinoma patients^27^, and II trials (NCT04225117; NCT04754191 in metastatic, castration-resistant prostate cancer) are ongoing across multiple tumor entities, mostly independent of Nectin-4 status, while selected studies investigate EV or other Nectin-4–targeted approaches specifically in Nectin-4–overexpressing tumors (NCT05097599). Novel Nectin-4–directed therapies, including ADCs, second generation Bicycle^®^ peptides that are covalently bound to MMAE (NCT04561362) or CD137 (NCT05163041), as well as fourth-generation CAR-T cells that jointly target Nectin-4 and FAP (NCT03932565), are currently in clinical development.

Key limitations of our study include the retrospective design, small cohort size, use of tissue microarrays, and the inherent subjectivity of immunohistochemical scoring.

In conclusion, although the prognostic relevance of Nectin-4 remains inconsistent across tumor types, our study provides the first evidence that membranous Nectin-4 expression is associated with poor prognosis in ccRCC. These findings support the potential role of Nectin-4 as both a prognostic biomarker and a therapeutic target in ccRCC, warranting further investigation.

## Data Availability

Data availability statement: The datasets used and/or analysed during the current study available from the corresponding author on reasonable request.
